# Insulin-like growth factor-1 (*IGF1*) genotype predicts breast volume after pregnancy and hormonal contraception and is associated with circulating IGF-1 levels: implications for risk of early-onset breast cancer in young women from hereditary breast cancer families

**DOI:** 10.1038/sj.bjc.6602389

**Published:** 2005-03-01

**Authors:** H Jernström, T Sandberg, E Bågeman, Å Borg, H Olsson

**Affiliations:** 1 Department of Oncology, The Jubileum Institute, Lund University Hospital, Lund SE-221 85, Sweden; 2 Department of Cancer Epidemiology, The Jubileum Institute, Lund University Hospital, Lund, Sweden

**Keywords:** *IGF1* genotype, IGF-1 levels, *BRCA1*, breast volume, premenopausal breast cancer, prospective cohort study

## Abstract

BRCA1/2 mutations predispose to early-onset breast cancer, especially after oral contraceptive (OC) use and pregnancy. However, the majority of breast cancers might be due to more prevalent low-penetrance genes, which may also modify the risk in *BRCA* mutation carriers. The absence of the *IGF1* 19-repeat allele has been associated with high insulin-like growth factor-1 (IGF-1) levels during OC use. High IGF-1 levels are linked to early-onset breast cancer and larger breast volumes in the general population. The goal of this study was to elucidate the relationships between *IGF1* genotype, early-onset breast cancer, breast volume, circulating IGF-1 levels and OC use in a prospective cohort of 258 healthy women ⩽40 years old from high-risk breast cancer families. All women completed a questionnaire including information on reproductive factors and OC use. We measured the height, weight, breast volumes and plasma IGF-1 levels. IGF-1 levels were similar among parous and nulliparous women not using OCs. In all, 13% had no *IGF1* 19-repeat allele. There was an interaction between *IGF1* genotype and OC use on IGF-1 levels (*P*=0.026) in nulliparous women and another interaction between *IGF1* genotype and parity on breast volume (*P*=0.01). Absence of the 19-repeat allele was associated with high IGF-1 levels in nulliparous OC users and with larger breast volumes in parous women and OC users. Incident breast cancers were also more common in women without the 19-repeat allele (log rank *P*=0.002). Our results suggest that lack of the *IGF1* 19-repeat allele modifies IGF-1 levels, breast volume and possibly early-onset breast cancer risk after hormone exposure in young high-risk women.

Early-onset breast cancer is a devastating disease for young women and their families. Several genes predisposing to early-onset breast cancer have been identified, most notably *BRCA1* and *BRCA2*. Carriers of the *BRCA1* and *BRCA2* mutations face a lifetime risk of breast cancer of over 80% (Easton *et al*, 1995; Tonin *et al*, 1995; Ford *et al*, 1998), although lower numbers have been reported (Streuwing *et al*, 1997; Antoniou *et al*, 2003). *BRCA1* and *BRCA2* promote differentiation and counteract proliferation during pregnancy and periods of concomitant oestrogen and progesterone administration (Marquis *et al*, 1995; Rajan *et al*, 1996), such as during oral contraceptive (OC) use. Both pregnancy and OC use increase the risk of breast cancer among carriers of *BRCA1* and *BRCA2* mutations (Jernström *et al*, 1999; Narod *et al*, 2002). In and of itself, teenage OC use has been associated with a small increase in early-onset breast cancer during use and up to 10 years following cessation of use in the general population (Collaborative Group on Hormonal Factors in Breast Cancer, 1996). Neither the presence of the *BRCA* mutations alone, nor the use of OCs, nor the combination of the two are sufficient to predict which women will develop early-onset breast cancer. It is likely that more prevalent low-penetrance genes in combination with environmental exposures modify breast cancer risk in *BRCA1* and *BRCA2* mutation carriers as well as contribute to breast cancer risk among women in the general population.

Insulin-like growth factor-1 (IGF-1) is essential for the normal development of the female breast both during puberty and pregnancy, along with various hormones, including oestrogen, progesterone and prolactin (Dickson, 1996). The IGF system plays a key role in regulating cell proliferation and apoptosis (Stewart and Rotwein, 1996). Insulin-like growth factor-1 levels inscribe a gene expression profile for angiogenic factors and cancer progression in breast epithelial cells (Oh *et al*, 2002). In the general population, high IGF-1 levels have been linked to premenopausal (Hankinson *et al*, 1998), but not to postmenopausal, breast cancer (Jernström and Barrett-Connor, 1999). Parous women in the general population have lower IGF-1 levels than nulliparous women (Holmes *et al*, 2002); after pregnancy, the risk of breast cancer is transiently increased, and is then lower for a period extending into the postmenopausal years (Beral and Reeves, 1993). It is possible that the decrease in IGF-1 levels in parous women accounts partially for the protective effect of parity against breast cancer.

The levels of circulating IGF-1 have also been linked to breast volume. High IGF-1 levels are associated with larger breast volumes in nulliparous women who are not using OCs (Jernström and Olsson, 1997). It has been observed that among young women who underwent hormonal breast augmentation with oestrogen, only those who responded with a significant increase in IGF-1 levels had substantial increases in breast volume (Hartmann *et al*, 1998). Increased levels of IGF-1 are also linked to increased breast density in premenopausal, but not postmenopausal, women (Byrne *et al*, 2000). Increased breast density after hormonal exposure is associated with increased risk of subsequent breast cancer (Boyd *et al*, 2001) and it has been hypothesised that increased breast volume during hormonal exposure may be a surrogate marker for increased breast cancer risk (Olsson, 1990).

The promoter region of the *IGF1* gene contains a CA-repeat sequence, which ranges from 12 to 23 repeats (Rosen *et al*, 1998). Most white women have at least one copy of the 19-repeat allele (Jernström *et al*, 2001b), but the frequency of the 19-repeat allele varies between ethnic groups (Jernström *et al*, 2001a). Insulin-like growth factor-1 levels decrease in most white OC users compared with white non-users (Jernström and Olsson, 1994). However, the absence of the common 19-repeat allele in the *IGF1* gene is associated with high IGF-1 levels during OC use in nulliparous women (Jernström *et al*, 2001a, 2001b). The risk of early-onset breast cancer after teenage OC use varies considerably between ethnic groups (Marcus *et al*, 1999), and appears to correlate with the relative frequencies of the absence of the 19-repeat allele among these ethnic groups (Jernström *et al*, 2001a). In addition, the absence of the 19-repeat allele appears to mitigate the normal age-related decline in IGF-1 levels (Rietveld *et al*, 2003). To our knowledge, no one has studied the interaction between the absence of the 19-repeat allele and parity on IGF-1 levels and subsequent breast cancer risk.

We have hypothesised that the elevation in IGF-1 levels, brought on by pregnancy or exogenous hormones (in particular teenage OC use) in the context of an absent 19-repeat allele, may play an important role in the pathogenesis of early-onset breast cancer and lead to an increase in breast volume (Figure 1). According to this hypothesis, the absence of the 19-repeat allele in and of itself may be a marker of increased early-onset breast cancer risk after pregnancy and OC use, and may be especially useful among *BRCA* mutation carriers where early-onset breast cancer is common. The aims of this study were to elucidate the relationships between *IGF1* genotype, early-onset breast cancer, breast volume, circulating IGF-1 levels, pregnancy and OC use in a prospective cohort of healthy premenopausal women from high-risk breast cancer families.

## MATERIALS AND METHODS

### Study population

To be eligible for participation, the women had to belong to high-risk breast cancer families and be either a known *BRCA1* or *BRCA2* mutation carrier or a first- or second-degree relative of a breast cancer case or known male or female *BRCA1* or *BRCA2* mutation carrier with or without breast cancer. We considered a family to be at high risk for breast cancer if three women had been diagnosed with breast cancer and at least one of these was below age 50 years at diagnosis, or two women and at least one was below age 40 years at diagnosis or one woman was diagnosed with breast cancer prior to age 30 years. Participants had to be 40 years of age or younger and menstruating. Exclusion criteria were a previous prophylactic mastectomy or a bilateral oophorectomy or any type of cancer diagnosis at the time of enrolment between 1996 and 2002. In all, 258 young women from breast cancer high-risk families volunteered to participate in this study. Potential participants were identified from charts and pedigrees from the Lund Oncogenetic Clinic. Individuals who themselves had been to the clinic were contacted, first by a letter including brief information on the study, then by phone. In cases where the index individuals were not eligible, they were asked whether they were willing to inform relatives of the study and then inform us as to whether we might contact their relatives directly. The ethical committee at the Lund University approved the study.

### Data collection

A letter including an extensive epidemiologic questionnaire and a written consent form was mailed to women who verbally agreed to participate. The questionnaire included questions on reproductive factors, such as age at menarche, menstrual cycle length, age at full-term pregnancies, duration of breast-feeding for each child, the use of OCs and other medications, smoking habits, diet, coffee consumption, physical exercise, etc. Other questions included the number of older siblings, the age of the mother and the age of the father of the woman when she was born. A trained research nurse collected blood samples and body measurements during menstrual cycle days 5–10 and again 5–10 days before the predicted onset of the following menstrual period. All women were asked to call back with the date of the first day of their next menstrual period. Body measurements included height, weight, waist and hip circumferences, and breast volume. Breast volumes were measured while the woman was on her hands and knees with the breasts hanging down. The volume was approximated to a pyramid (base area × height/3). Total breast volume, that is, the sum of the right and left breast volumes, was calculated for each visit. Initially, we only measured one of the breasts and for the first woman total breast volume is missing for both visits and for the next three women total breast volume is missing for one visit. The plasma and blood cells were separated and frozen at minus 70°C at our laboratory at the Department of Oncology, Lund.

### Mutation testing

Mutation testing of the *BRCA1* and *BRCA2* genes was not performed as part of this study. BRCA1 and BRCA2 mutation testing is offered at the Oncogenetic clinic at the Department of Oncology in Lund if an individual has at least three first-degree relatives with breast cancer and one diagnosed before age 50; two first-degree relatives with breast cancer and one diagnosed before age 40; or one first-degree relative with breast cancer diagnosed before age 30. Women are usually not offered testing before age 25 years. *BRCA* gene mutation carriers included only those with confirmed deleterious alterations, that is, nonsense or frameshift indel mutations that cause protein truncation, or known disease-associated missense mutations.

The participating women were classified into eight different categories:

1. *BRCA1* mutation carriers (*n*=20);
2. non-*BRCA1* mutation carriers from a family with a known *BRCA1* mutation (*n*=47);
3. untested women from a family with a known *BRCA1* mutation (*n*=17);
4. *BRCA2* mutation carriers (*n*=7);
5. non-*BRCA2* mutation carriers from a family with a known *BRCA2* mutation (*n*=6);
6. untested women from a family with a known *BRCA2* mutation (*n*=2);
7. women belonging to a family where no *BRCA1* or *BRCA2* mutations could be detected, that is, members of a ‘non-*BRCA1/2* family’ (*n*=113);
8. women from untested high-risk families (*n*=46). These families were untested for various reasons, for example, all breast and ovarian cancer cases were deceased or refused testing.

### Follow-up

Women were followed until the development of a first breast cancer according to the regional cancer registries, until the date of a self-reported prophylactic mastectomy or until March 31, 2003, whichever came first. No woman had undergone a prophylactic oophorectomy before undergoing a prophylactic mastectomy. The report rate of cancer diagnoses to the Swedish cancer registries is close to a 100%. The clinical follow-up of high-risk women includes annual mammograms, ultrasounds and MRIs of the breasts in addition to a physical examination and annual follow-ups of the ovaries by ultrasound, CA-125 and a gynecological examination.

### DNA testing

Genomic DNA was extracted from 300 *μ*l peripheral blood using Wizard, Genomic DNA Purification Kit, Promega. The polymorphism in the *IGF1* gene is a tandem dinucleotide repeat, ranging in size from 12 to 23 repeats. PCR primers 5′-GCTAGCCAGCTGGTGTTATT and 5′-GTTTCTTACCACTCTGGGAGAAGGGTA were used, where the forward primer was fluorescently labelled with FAM (MWG-Biotech AG).

PCR was performed in 15-*μ*l reactions using 25 ng DNA, 0.4 *μ*M of each primer, 0.1 mM of each deoxynucleotide (Amersham Biosciences, Buckinghamshire, UK), 5% DMSO (Sigma, St Louis, MO, USA), 2.5 mM MgCl_2_ (Applied Biosystems, Foster City, CA, USA), 1 × PCR Gold buffer (Applied Biosystems) and 1 U AmpliTaq Gold (Applied Biosystems).

The PCR product was analysed in an ABI3100 Genetic Analyzer (Applied Biosystems) and the results were evaluated using Genescan software. The number of repeats was determined by sequencing samples of varying sizes (Big Dye, Terminator Cycle Sequencing, Applied Biosystems) and using them as standards in fragment size analysis.

### Insulin-like growth factor-1 analyses

Insulin-like growth factor-1 was analysed in EDTA plasma using the radio-immuno assay (RIA) method at the Endocrinological laboratory at the Karolinska Hospital, Stockholm, Sweden, as previously described by Bang *et al* (1991). Acid ethanol extraction followed by cryo-precipitation was used to separate insulin-like binding proteins (IGFBPs) from IGF-1 and decrease the interference of IGFBPs in the IGF-1 RIA. Recombinant human IGF-1 (Kabi-Pharmacia, Stockholm, Sweden) was used as standard. The international IGF-1 standard was delivered by the National Institute of Biological Standards and Control, London, Great Britain. The intra-assay variation was 4% and the inter-assay variation was 11%. The limit of detection was 6 *μ*g l^−1^.

### Data analyses

The statistical software SPSS 10.0.7 was used for all statistical analyses. Breast volumes and body weights were not normally distributed and the values were transformed using the natural logarithm to obtain better distribution. Spearman rank correlation coefficients were used for correlations. Mann–Whitney *U*-test was used to compare the body weight between parous and nulliparous women. Chi-square was used to compare the frequency of the absence of *IGF1* 19-repeat allele between known BRCA1 mutation carriers and other women. Multivariate linear regression was used to compare circulating IGF-1 levels in relation to genotype, parity and use of hormonal contraceptives. A log-rank test was used to analyse the risk of breast cancer in relation to *IGF1* genotype. Cox regression was used to analyse the hazard of developing breast cancer in relation to *IGF1* genotype adjusting for other factors. As the study was based on *a priori* hypotheses, that is, that the *IGF1* genotype was associated with an idiosyncratic response to hormone exposure in young women (Jernström *et al*, 2001a, 2001b), no adjustment for multiple testing was required. We calculated the sample size needed to detect a minimum of 20% difference (±) in breast volume between nulliparous and parous women with and without the 19-repeat allele, with 80% power and an alpha value of 0.05, assuming a mean breast volume of 900 cm^3^ and a standard error of 250, and that 50% of the women were parous and equally distributed between the two genotypes. If the frequency of the absence of the 19-repeat allele were 10% we would need 250 women ,and if the frequency were 15% we would need 177 women. A *P*-value of <0.05 was taken to be significant. All *P*-values were two-sided.

## RESULTS

The characteristics of the 258 women at baseline are described in Table 1. Six women had developed cancer during follow-up. There were no significant differences in reproductive factors, body sizes or use of hormonal contraceptives between women with and without breast cancer at follow-up. In all, 254 women agreed to undergo genotyping of the *IGF1* gene. The *BRCA1* and *BRCA2* mutation status of the women with and without breast cancer is described in Table 2; four cases were known *BRCA1* mutation carriers and two cases came from untested families and have not undergone testing themselves. The allele frequency distribution of the *IGF1* gene is described in Table 3; absence of the *IGF1* 19-repeat allele was more common among the women with breast cancer (66.7%) than among the women without breast cancer (11.7%). Total breast volume measurements, taken 5–10 days before the predicted onset of the next menstrual period, were available for 240 women who were not currently breast-feeding and who had not undergone previous breast augmentation or reduction surgeries.

Breast volume was strongly correlated with body weight (*r*_s_=0.68; *P*<0.001) and the waist-to-hip ratio (*r*_s_=0.50; *P*<0.001), but not with height (*r*_s_=0.15; *P*=0.08). Parous women were on average 5.4 kg heavier than nulliparous women (*P*=0.001). The weight-adjusted breast volumes in nulliparous and parous women depended on the *IGF1* genotype and current hormonal contraceptive status. In all, 87% of the women carried at least one *IGF1* 19-repeat allele. Women who lacked the *IGF1* 19-repeat allele were nonsignificantly lighter than women with at least one 19-repeat allele (*P*=0.15, adjusted for parity), while no effect on height was seen. There was a strong interaction between parity and *IGF1* genotype on breast volume (*P*=0.01). We therefore stratified the women depending on the absence or presence of the 19-repeat allele. Among the 209 women with at least one 19-repeat allele, the weight-adjusted breast volume was significantly smaller in parous women compared with nulliparous women (*P*=0.02) and nonsignificantly smaller in current oral or hormonal contraceptive users (*P*=0.06). Conversely, among the 30 women with no 19-repeat allele, the weight-adjusted breast volumes were nonsignificantly larger in parous than in nulliparous (*P*=0.06), and also nonsignificantly larger in current OC users (*P*=0.12).

We collected plasma for studying circulating levels of IGF-1 at two time-points during the menstrual cycle from each woman, 5–10 days after onset of the menstrual period and again 5–10 days prior to the predicted onset of the next menstrual period, that is, during menstrual cycle days 18–23 in most women. Insulin-like growth factor-1 levels were missing during cycle days 5–10 for one woman. For several of the hormonal contraceptive users, the samples obtained during cycle days 5–10 were collected during their pill-free week. However, the second samples were obtained during cycle days 18–23, when the women were taking active pills. We excluded the five women who were currently breast-feeding at the time of blood collection from the analyses.

Age was correlated with IGF-1 levels both during cycle days 5–10 (*r*_s_=0.51; *P*<0.001) and during cycle days 18–23 (*r*_s_=0.37; *P*<0.001). There were no major differences in age decline in circulating IGF-1 levels in the samples collected during cycle days 5–10 between women with or without the IGF-1 19-repeat allele (*r*_s_=0.50 and 0.52, respectively), while the age decline in the samples obtained during cycle days 18–23 was larger among women with than without the 19-repeat allele (*r*_s_=0.39 and 0.30, respectively).

**Figure 2:**
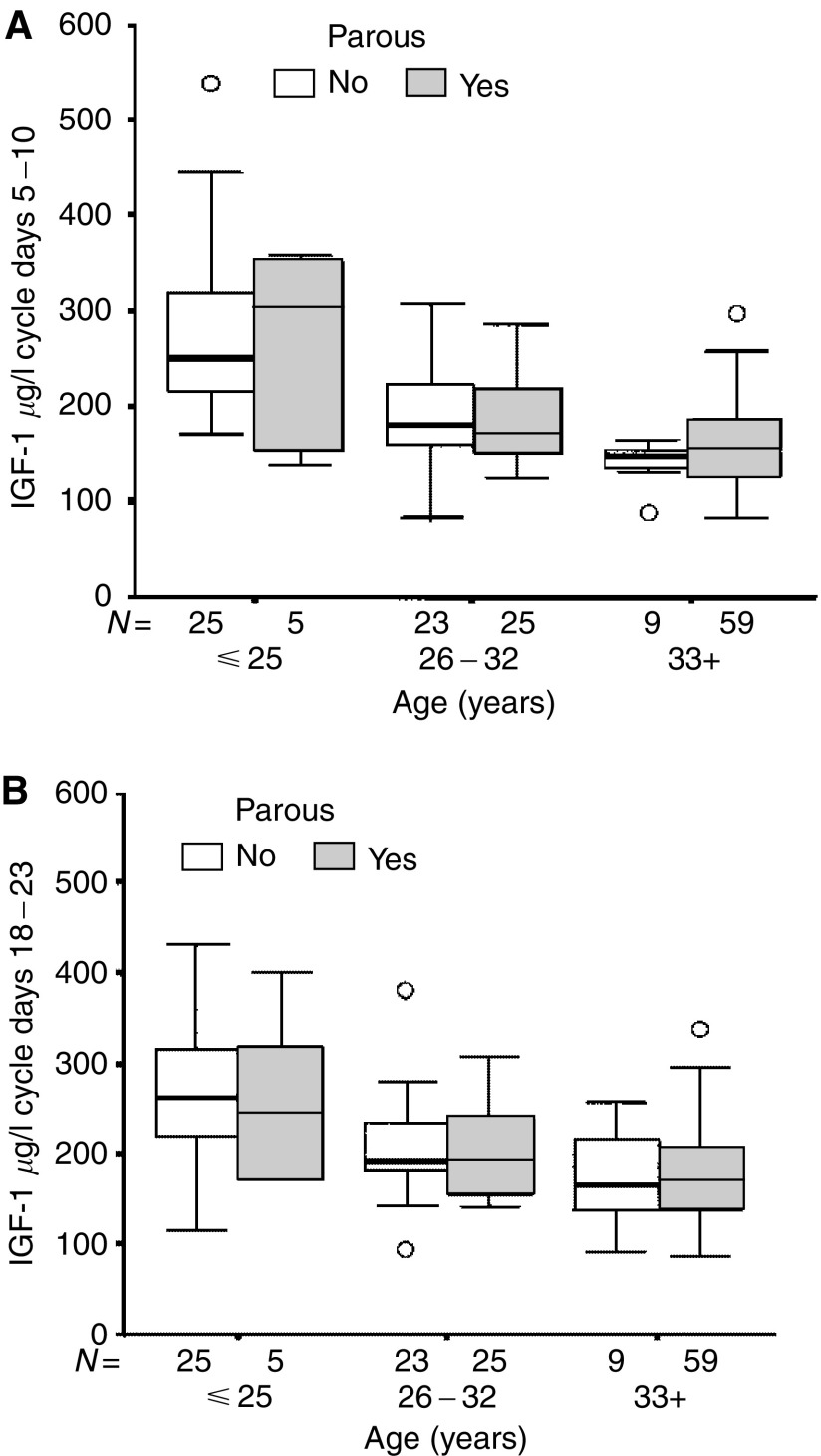
(**A**, **B**) show that there were no significant differences in median IGF-1 levels between parous and nulliparous women not currently using any hormonal contraception in the samples obtained during cycle days 5–10 (*P*=0.57) or in the samples obtained during cycle days 18–23, that is, 5–10 days prior to the predicted onset of the next menstrual period (*P*=0.49). The horizontal line in the box indicates the median value, the box boundaries the 25th and 75th percentiles, and the capped bars the 10th and the 90th percentiles. The number of women in each group is indicated. Circles indicate outliers with values between 1.5 and 3 box lengths from the upper or lower edge of the box. Asterisks indicate extreme outliers with values more than 3 box lengths from the upper or lower edge of the box. The box length is the interquartile range.

Parity and current hormonal contraception had no significant impact on the age-adjusted IGF-1 levels obtained during cycle days 5–10 (*P*=0.39 and 0.67, respectively). During cycle days 18–23, there was an interaction between parity and current hormonal contraception on IGF-1 levels (*P*=0.05). The age-adjusted IGF-1 levels during cycle days 18–23 were similar among parous and nulliparous women not using hormonal contraception (*P*=0.49, Figure 2). Current hormonal contraception was strongly associated with lower age-adjusted IGF-1 levels (*P*=0.00003) among nulliparous women, while no such effect was seen among parous women (*P*=0.35). Among hormonal contraceptive users, parous women had nonsignificantly higher circulating IGF-1 levels compared with nulliparous hormonal contraceptive users (*P*=0.11). The results remained essentially the same if women who were using other types of hormonal contraception than combined OCs were excluded.

**Figure 3:**
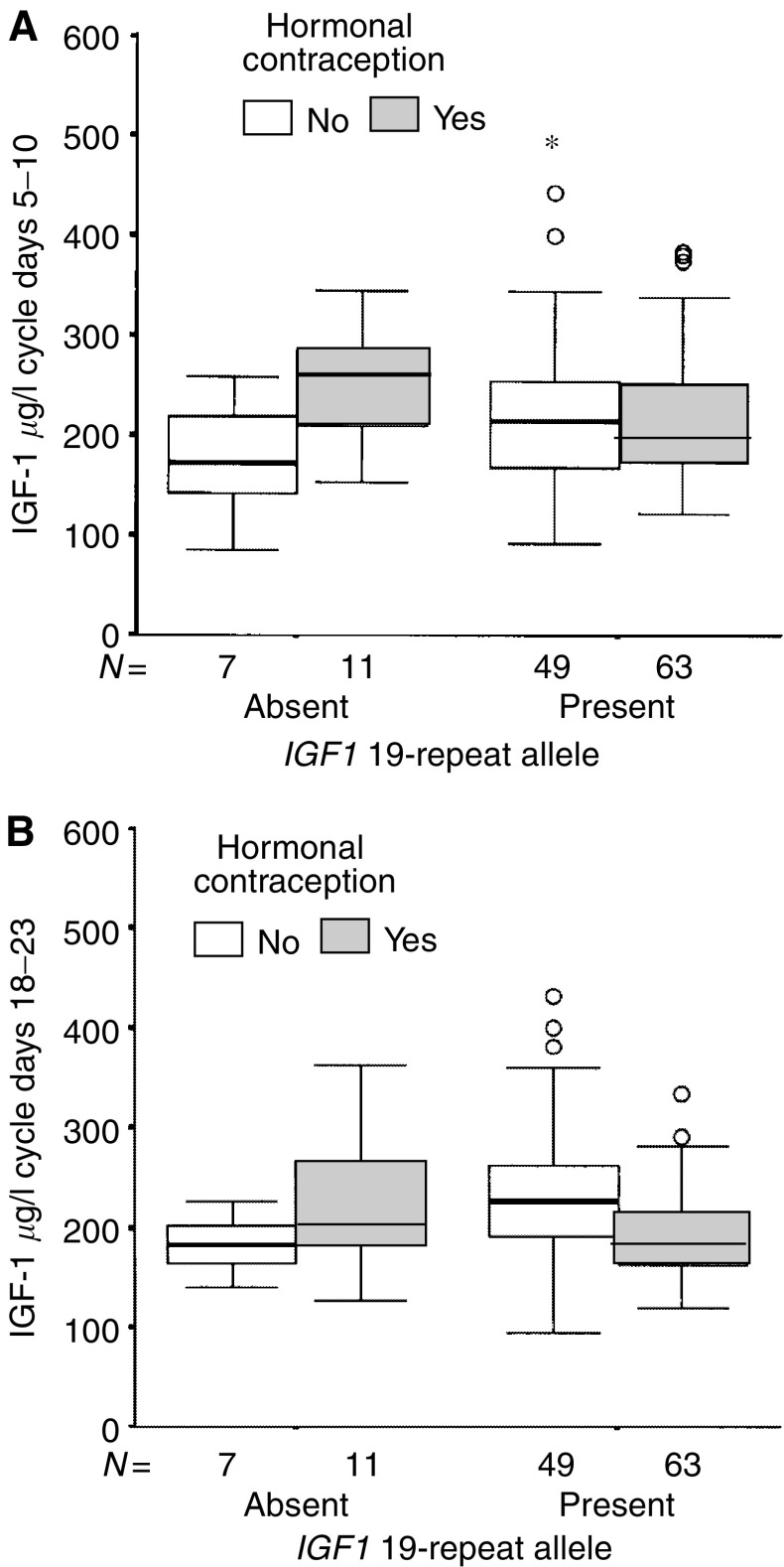
(**A**, **B**) show that the *IGF1* genotype modified the effect of hormonal contraceptives on the IGF-1 levels in nulliparous women. The interaction between IGF-1 genotype and hormonal contraception was nonsignificant during cycle days 5–10 (*P*=0.13) and significant during cycle days 18–23, that is, 5–10 days prior to the predicted onset of the next menstrual period (*P*=0.026). The horizontal line in the box indicates the median value, the box boundaries the 25th and 75th percentiles, and the capped bars the 10th and the 90th percentiles. The number of women in each group is indicated. Circles indicate outliers with values between 1.5 and 3 box lengths from the upper or lower edge of the box. Asterisks indicate extreme outliers with values more than 3 box lengths from the upper or lower edge of the box. The box length is the interquartile range.

The *IGF1* genotype modified the effect of hormonal contraceptives on the IGF-1 levels during cycle days 18–23 among nulliparous women, and there was a significant interaction between *IGF1* genotype and hormonal contraception on IGF-1 levels (*P*=0.026, Figure 3). Nulliparous hormonal contraceptive users with at least one copy of the 19-repeat allele had significantly lower age-adjusted IGF-1 levels than non-users (*P*=7 × 10^−6^), but not hormonal contraceptive users lacking the 19-repeat allele (*P*=0.81), confirming previous studies of an idiosyncratic response in circulating IGF-1 levels to hormonal contraception among nulliparous women lacking the *IGF1* 19-repeat allele. The results remained essentially the same for nulliparous women if women who were using hormonal contraception other than combined OCs were excluded. Among parous women, no significant effect on age-adjusted IGF-1 levels from hormonal contraceptive use was seen, independent of *IGF1* genotype. The results remained essentially the same if women who were using hormonal contraception other than combined OCs were excluded.

The *IGF1* genotype did not significantly modify the effect of pregnancy on circulating IGF-1 levels among women not using hormonal contraception and who were not currently breast-feeding. No significant difference between parous and nulliparous women was seen irrespective of the absence or presence of the 19-repeat allele.

**Figure 4:**
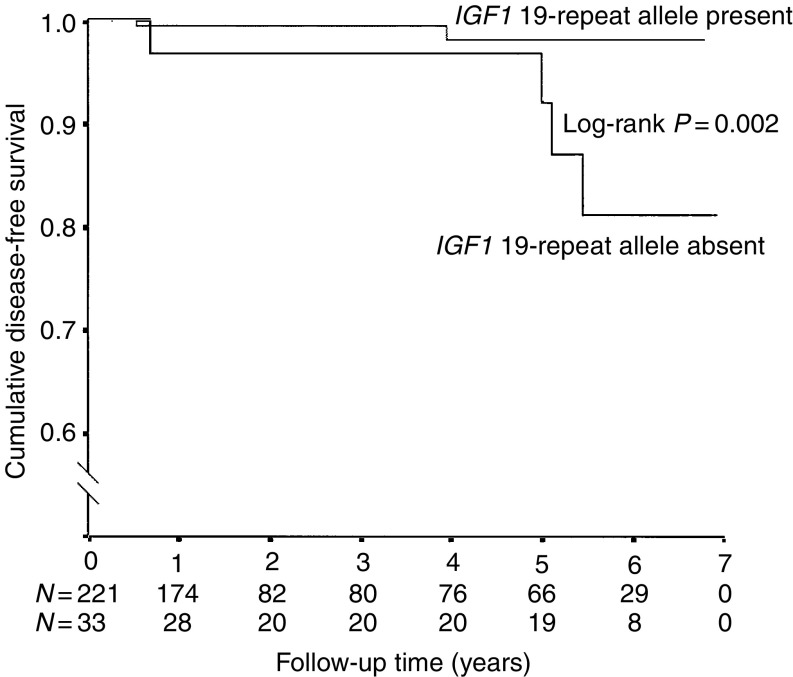
The figure shows the cumulative breast-cancer-free survival after study entry in women with and without the *IGF1* 19-repeat allele. The difference between the two groups was significant (log-rank *P*=0.002). Follow-up was censored at the time of prophylactic mastectomy without detection of breast cancer or on March 31, 2003. Please note the broken *Y*-axis. The number of women at each time point is indicated.

To date, six women have been diagnosed with breast cancer, three with bilateral disease. The median follow-up time of the cohort was 1.4 years (range 0.07–6.93 years). Two of the breast cancers were diagnosed at the time of prophylactic mastectomy. In all, 14 other women reported having undergone prophylactic mastectomies after inclusion in this study, but no cancers were detected at the time of surgery. Four cases were known BRCA1 mutation carriers and two cases belonged to untested families and have chosen to not undergo testing themselves after their cancer diagnoses. The median age at diagnosis was 38.5 years (range 28–44 years). All cases had started OC use during their teenage years and only one had never given birth. Absence of the *IGF1* 19-repeat allele was associated with breast cancer during follow-up (log-rank *P*=0.002, Figure 4). We then restricted the analysis to include only women who had been exposed to OCs during their teenage years. The age-adjusted hazard ratio for women who had started OC use before age 20 years and who had no copy of the 19-repeat allele compared to women with at least one copy of the 19-repeat allele was 10.2 (95% CI 1.85–56.7; *P*=0.007). In the second model, we included only parous women. The age-adjusted hazard ratio for parous women lacking the 19-repeat allele was 6.62 (1.05–41.67; *P*=0.04) compared with parous women with at least one copy of the 19-repeat allele.

A large proportion of the women are still untested for *BRCA1* and *BRCA2* mutations, mostly because of their young age or because no affected family member could be tested. Absence of the 19-repeat allele of the *IGF1* gene was more often found in known *BRCA1* mutation carriers than in other women (30.0 *vs* 11.5%; *P*=0.018) and the two variables could therefore not be examined in the same model. Our data suggest that *BRCA1* mutation carriers who also lack the *IGF1* 19-repeat allele may be at a higher risk for early-onset breast cancer than *BRCA1* mutation carriers with presence of the 19-repeat allele. Among the 20 women with known *BRCA1* mutations, six were lacking the 19-repeat allele. Of these six women, two have undergone prophylactic mastectomy, three have been diagnosed with breast cancer and one is still healthy. Among the 14 women with at least one copy of the 19-repeat allele, six have undergone prophylactic mastectomy, one has been diagnosed with breast cancer and seven are still healthy.

As an additional control group, we also performed *IGF1* genotyping on 86 women with available DNA from the South Swedish *BRCA1* mutation families who did not carry the mutation in their respective families and who did not have breast cancer at the time of testing. These women were between 20 and 91 years old at the time of testing. Three of the 32 women (9.4%) who were 40 years or younger at the time of testing lacked the 19-repeat allele, and two of the 54 women (3.7%) who were 41 years or older at the time of *BRCA1* mutation testing lacked the 19-repeat allele. When this additional control group and the 45 *BRCA1* negative women in the present study were considered together, the frequency of the absence of the 19-repeat allele was 8.4% among all BRCA1-negative women in the South Swedish region with available DNA. For women aged 40 years or younger at testing, the frequency was 11.7%.

## DISCUSSION

The main findings of this study were that the absence of the common *IGF1* 19-repeat allele was associated with larger breast volumes in parous women and in hormonal contraceptive users, after adjustment for body weight, and secondly that parity was not associated with a decrease in circulating IGF-1 levels contrary to the report based on women in the general population (Holmes *et al*, 2002). We also confirmed our previous reports based on healthy nulliparous Canadian women (Jernström *et al*, 2001a, 2001b) that the absence of the *IGF1* 19-repeat allele was associated with an idiosyncratic response on circulating IGF-1 levels to hormonal contraception among nulliparous women. Our preliminary analyses suggest an increased risk of early-onset breast cancer risk after hormonal exposure, such as teenage hormonal contraception or a pregnancy, in women lacking the *IGF1* 19-repeat allele.

High IGF-1 levels are associated with increased risk for premenopausal breast cancer (Hankinson *et al*, 1998). They inscribe a gene expression profile for angiogenic factors and cancer progression in breast epithelial cells (Oh *et al*, 2002) and accelerate the progression of precancerous changes to invasive lesions. Thus, on the basis of epidemiological and experimental studies, a decrease in circulating IGF-1 levels may help reduce the risk of breast cancer, especially among members of high-risk families (Furstenberger and Senn, 2002). As for postmenopausal breast cancer, neither the levels of circulating IGF-1 (Jernström and Barrett-Connor, 1999) nor the *IGF1* genotype appear to affect the risk (Missmer *et al*, 2002; DeLellis *et al*, 2003). The frequency of the absence of the 19-repeat allele varies greatly between ethnic groups and is highest among African-American women (Jernström *et al*, 2001a; DeLellis *et al*, 2003). Missmer *et al* (2002) found no interaction between the absence of the 19-repeat allele and overall breast cancer risk. However, they did not match their cases and controls for ethnic background, which may have affected their results. They also did not find any association between this genotype combined with ever OC use and breast cancer, but they did not examine the effect of teenage OC use, and the women included in that study were older than in our study, range 43–69 years, and were thus unlikely to be diagnosed with OC-associated early-onset breast cancer. The effect of OCs on breast cancer is generally thought to be limited to early-onset breast cancer, for example, prior to age 35 or 40 years of age (Collaborative Group on Hormonal Factors in Breast Cancer, 1996). Neither the study by Missmer *et al* (2002) nor the study by DeLellis *et al* (2003) examined the possibility that the absence of the *IGF1* 19-repeat allele is a latent polymorphism that only gives rise to an increased risk of early-onset breast cancer in combination with teenage OC use, and possibly pregnancy, but does not affect late-onset breast cancer. A recent case–control study of breast cancer in twins suggested that the breast tissue in women with a certain genotype might show an unusual sensitivity to pubertal hormones and an absence of linkage to hormonal milestones later in life (Hamilton and Mack, 2003). Teenage OC use has been associated with a several fold increased risk of early-onset breast cancer compared with use commenced after age 20 years (Olsson *et al*, 1989).

Twin studies (Kao *et al*, 1994; Harrela *et al*, 1996) have shown that about 50% of the inter-individual variability in circulating IGF-1 levels is genetically determined. The *IGF1* polymorphism is located in the promoter region of the *IGF1* gene (Rosen *et al*, 1998). This microsatellite polymorphism is located one kilobase upstream from the *IGF1* transcription start site (Rotwein *et al*, 1986; Weber and May, 1989) and contains specific regulatory elements (McCarthy *et al*, 1997). *In vitro* studies of rat osteoblasts have shown a 100–300-fold difference in their ability to suppress the stimulatory effect of prostaglandin E_2_ in response to 17-alpha and 17-beta estradiol when they bind to the oestrogen response elements within the *IGF1* promoter region (McCarthy *et al*, 1997). The *IGF1* polymorphism appeared to have no impact on IGF-1 levels in nulliparous women who are not using OCs, while women with no copies of the *IGF1* 19-repeat allele demonstrated high IGF-1 levels during OC use (Jernström *et al*, 2001a, 2001b). The present study confirmed our previous findings that hormonal contraceptives were associated with decreased circulating IGF-1 levels only among nulliparous women with presence of the 19-repeat allele. The difference in IGF-1 levels among OC users with and without the 19-repeat allele suggests that this allele may be associated with a conformational change in the region of the IGF-1 promoter, possibly involving the oestrogen response element. However, we did not observe the same effect among parous women in the current study.

The women included in our cohort were selected on the basis of the fact that they belonged to high-risk breast cancer families with several cases of breast or ovarian cancer. The penetrance of the *BRCA1* and *BRCA2* genes varies according to the selection of the families (Easton *et al*, 1995; Tonin *et al*, 1995; Antoniou *et al*, 2003). It is therefore likely that other modifying genetic variants segregate at a high frequency in the current cohort, because it is based on a selection of volunteers from families with multiple cases of cancers. The percentage of women without a copy of the 19-repeat allele was higher in the present cohort (13%) than among a group of healthy white women from the general population living in Toronto, Canada (8%) (Jernström *et al*, 2001b). As an extra control group, we also performed additional *IGF1* genotyping on *BRCA1*-negative women belonging to the South Swedish *BRCA1* families with available DNA, who did not have breast cancer at the time of mutation testing. In total, 8.4% of the *BRCA1* non-carriers lacked the 19-repeat allele. We chose to genotype non-carriers from the same families as the women participating in the study, because these women belong to the same ethnic and socioeconomic background and live in the same geographic area as the participants and are thus optimal controls. Others have reported similar frequencies for the absence of the 19-repeat allele among Caucasians ranging between 6.4 and 11.6% (Vaessen *et al*, 2001; DeLellis *et al*, 2003). The absence of the *IGF1* 19-repeat allele was found at a higher frequency among known BRCA1 mutation carriers (30%) compared with other women (11.5%) in the present study. Conversely, all women who belonged to families with BRCA2 mutations carried at least one copy of the 19-repeat allele in the present study. Abramovitcha *et al* (2003) have presented evidence suggesting a link between the IGF-1 system and *BRCA1*, and reported that the transcription of the IGF-1 receptor gene in breast cancer-derived cell lines is under the inhibitory control of BRCA1. Others have reported that the *IGF1* genotype is linked to a low birth weight (Arends *et al*, 2002) and we have previously reported low birth weight among BRCA1 carriers compared with non-carriers from BRCA1 families (Jernström *et al*, 1998), although no IGF-1 genotyping was performed in the latter study. The link between the IGF-1 system and the *BRCA1* gene clearly warrants further study, and we are currently undertaking *IGF1* genotyping of all *BRCA1* carriers to elucidate whether the absence of the 19-repeat allele is more frequent in mutation carriers than non-carriers. Further studies are needed to elucidate whether the combination of a *BRCA1* mutation and lack of the 19-repeat allele renders *BRCA1* carriers especially susceptible to early-onset breast cancer after OC exposure (Narod *et al*, 2002) and pregnancy (Jernström *et al*, 1999). Due to the limitation of our sample size, we cannot exclude the possibility that other repeat lengths of the *IGF1* gene are associated with breast cancer.

This is an early report from a prospective cohort of young healthy women from high-risk breast cancer families. So far six participants have been diagnosed with breast cancer, three with bilateral disease. The median time of follow-up is now 1.4 years or 776 person-years. This gives an annual incidence in our cohort of 0.77%. This overall annual incidence rate is lower than in other cohorts of high-risk women consisting of BRCA1 and BRCA2 carriers (Easton *et al*, 1995; Tonin *et al*, 1995; Ford *et al*, 1998). However, the present cohort includes 53 women who are known to not carry the respective BRCA1 or BRCA2 mutations that segregate in their families, as well as 113 women from non-BRCA1/2 families and 46 women from untested families. Four of the six cases are known BRCA1 mutation carriers, while two cases were untested, and we cannot exclude the possibility that they also carry BRCA1 or BRCA2 mutations. None of the six cases shared any familial relationship as far as we know. In general, we have extended the pedigrees as far back as possible, that is, usually two to four generations; in several cases, Swedish Registry Data have been used to confirm distant relatives. In addition to the two women whose cancers were detected during prophylactic mastectomy, 14 other women also reported to have undergone the procedure. These operations are self-reported and it is possible that other women have undergone the operation without notifying us. However, given that high-risk women undergo annual clinical examinations, it is unlikely that we missed more than one or two mastectomies, if any.

It would have been optimal if we had had mutation status on all participants in the study. However, in Sweden, BRCA1/2 mutation testing is not normally performed on women younger than 25 years of age, unless a family member has developed breast cancer prior to age 30 years. The risk of developing breast cancer prior to 25 is considered very low even for mutation carriers. We included women as young as 18 years old in the study and they must decide for themselves whether they want to get tested when they turn 25. Several of the untested women have chosen to be tested after inclusion in the study, but quite a few of the youngest women are still untested. For women belonging to untested families, we are unable to test the women until a family member with breast or ovarian cancer agrees to get tested. The two women from untested families who themselves got breast cancer have chosen to not undergo mutation testing at this point and we are prohibited by Swedish law to perform mutation testing against somebody's will.

In our study, the body-weight-adjusted breast volumes were larger in parous than in nulliparous women and in hormonal contraceptive users lacking the 19-repeat allele than in non-users, while the opposite was true for women carrying at least one copy of the 19-repeat allele and the interaction was highly significant. This suggests a differential response in the breast tissue to both pregnancy and exogenous hormone exposure depending on *IGF1* genotype. It also suggests increased breast epithelial proliferation after pregnancy, resulting in permanently larger breast volumes in women lacking the 19-repeat allele. Insulin-like growth factor-1 stimulates cell proliferation and reduces cell apoptosis (LeRoith and Roberts, 2003) and is associated with breast volume (Jernström and Olsson, 1997; Hartmann *et al*, 1998). Dupont and Page reported a higher risk of breast cancer with larger breast volumes, but only in women with proliferative breast disease (Dupont and Page, 1987). Conversely, Thurfjell *et al* (1996) reported small breast sizes to be associated with increasing breast risk through their association with high-risk parenchymal pattern among Swedish women undergoing mammography. However, Egan *et al* (1999) reported that only larger breast sizes in lean women prior to the first full-term pregnancy were associated with increased risk of breast cancer, and commented that studies that lacked an associated between breast size and breast cancer did not consider the effect modification by overall obesity. A full-term pregnancy is associated with lower levels of IGF-1 in the general population (Holmes *et al*, 2002), although it is unknown whether this also pertains to women with no copy of the 19-repeat allele. In the present study of high-risk women, we did not observe lower levels of IGF-1 in parous compared with nulliparous women irrespective of *IGF1* genotype, which is in contrast to the previous report (Holmes *et al*, 2002). Furthermore, each pregnancy up to three is associated with an increased risk of early breast cancer among BRCA1 and BRCA2 carriers (Jernström *et al*, 1999) and it is possible that this is partly mediated through the IGF-1 pathway. Based on our observation of a statistically significant interaction between parity and *IGF1* genotype on body-weight-adjusted breast volume, it is possible that this gene could be a marker of women at higher risk for pregnancy-associated breast cancers. Insulin-like growth factor-1 is one of the key growth factors in breast epithelial development (Hovey *et al*, 2002). We have previously reported an association between breast volume and IGF-1 levels in nulliparous women not using OCs (Jernström and Olsson, 1997). Hartmann *et al* (1998) have reported that only young women who responded with a significant increase in IGF-1 levels when they underwent hormonal breast augmentation with oestrogen demonstrated substantial increases in their breast volumes. The *IGF1* genotype was not analysed in that study. It would be optimal if we could measure breast sizes and IGF-1 levels before and after OC use and pregnancies in a large population-based cohort of women with known *IGF1* genotype to confirm whether the response to hormone exposure is dependent on the *IGF1* genotype. To our knowledge, the present study is the first to explore the association between *IGF1* genotype and breast volume.

There is no standard way of measuring breast volume. In this study, breast volume was measured and approximated to a pyramid for practical reasons. We have previously published data on breast volume measured by the same technique as in the present study in relation to exogenous and endogenous hormone exposure among 19–25-year-old healthy nulliparous women (Jernström and Olsson, 1997). The correlation between the measurements taken on cycle days 5–10 and 18–23 was high in the present study (*r*_s_=0.98; *P*<0.0001). Using brassiere cup size as a measurement of breast volume is a less satisfactory method, since different brands of brassieres differ in size. Furthermore, an increase in rib cage circumference results in a decrease in cup size. Thus, for two women with the same breast volume but different rib cage circumferences, the woman with the narrower rib cage would wear a larger cup size than the one with the wider rib cage. A more exact procedure may have been to use water displacement, which we actually tried, but it ended up being very messy and was actually less reproducible than the approximated pyramid. We only have breast measurements taken either before or after pregnancy, and serial measurements in each woman to confirm an actual increase or decrease in breast volume depending on *IGF1* genotype would have been preferable. Mammography or ultrasound would have allowed us to validate the amount of breast epithelium of each woman. However, because many women in our study were very young, not all of them have undergone mammography or ultrasound.

The main findings of this prospective study were that the absence of the *IGF1* 19-repeat allele was associated with larger breast volumes in parous women and hormonal contraceptive users and an idiosyncratic response to hormonal contraceptives among nulliparous women. An early analysis of new breast cancers in the cohort also supported our hypothesis that absence of the 19-repeat allele in combination with hormonal contraceptives may be associated with an increased risk of early-onset breast cancer. The absence of the *IGF1* 19-repeat allele may be a new biomarker of increased OC- and pregnancy-associated breast cancer, especially among BRCA mutation carriers where early-onset breast cancer is common. To our knowledge, this is the first time the effect of the *IGF1* genotype in combination with teenage OC use on early-onset breast cancer incidence has been explored. Our data are strong enough to tolerate up to four additional cases that all carry the common *IGF1* 19-repeat allele instead of the high-risk genotype, that is, absence of the 19-repeat allele, and still remain significant. However, this is an early report and whether the absence of the *IGF1* 19-repeat allele could be used as an additional marker to improve risk estimates for early-onset breast cancer during genetic counselling warrants confirmation in a larger cohort.

## Figures and Tables

**Figure 1:**
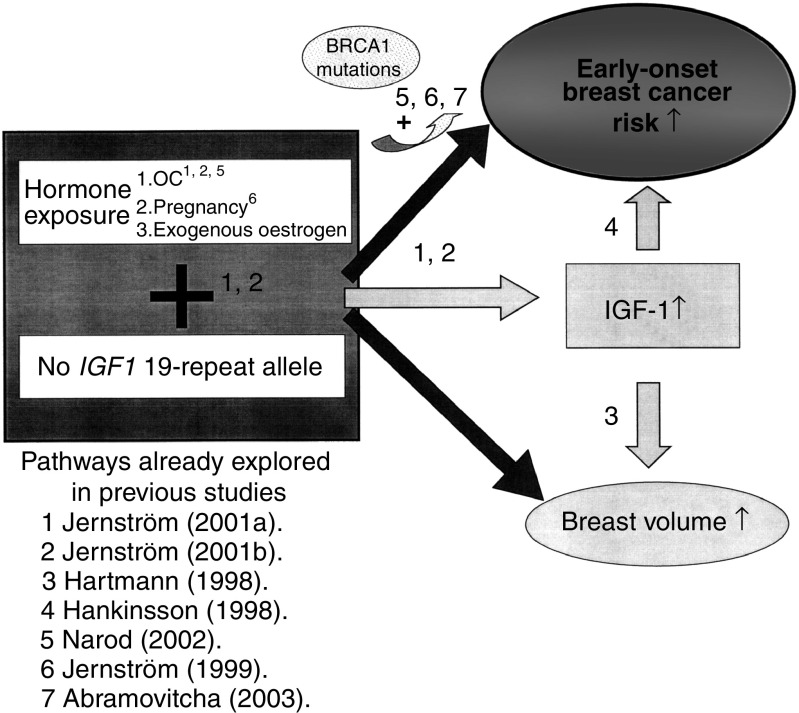
The figure shows the proposed mechanisms by which we hypothesise that absence of the *IGF1* 19-repeat allele in combination with hormone exposure would increase the breast volume and the risk of early-onset breast cancer. References to the steps that have been previously explored are indicted. The risk of early-onset breast cancer after hormone exposure may be partially modified through the IGF-1 pathway.

**Table 1:** Characteristics of women with and without breast cancer at follow-up

	**No breast cancer (*n*=258)**		**Breast cancer (*n*=252)**		**No breast cancer *vs* breast cancer (*n*=6)**		
**All women**	**Mean**	**(±s.d.)**	**Mean**	**(±s.d.)**	**Mean**	**(±s.d.)**	***P*-value**
Age at baseline	29.1	(±6.3)	29.1	(±6.4)	33.3	(±6.3)	0.10
Age at menarche	12.8	(±1.3)	12.8	(±1.3)	13.2	(±0.4)	0.06
Parous at baseline	126/258 (49%)		121/252 (48%)		5/6 (83%)		0.08
Age at first birth	24.8	(±4.0)	24.7	(±4.0)	26.0	(±3.7)	0.48
Smoker at baseline	58/257 (23%)		55/251 (22%)		3/6 (50%)		0.10
Oral contraceptives, ever	237/258 (92%)		231/252 (92%)		6/6 (100%)		0.46
Start age (years)	17.2	(±2.7)	17.2	(±2.7)	16.8	(±1.3)	0.74

*Hormonal contraception, baseline*							
OC	100/252 (39%)		99/252 (39%)		1/6 (17%)		
Levonova prog IUD	6/258 (2.3%)		6/252 (2.4%)		0/6 (0%)		
Norplant	2/258 (0.8%)		2/252 (0.8%)		0/6 (0%)		
Implanon	1/258 (0.4%)		1/252 (0.4%)		0/6 (0%)		

Height, cm	168	(±6.0)	168	(±6.0)	168	(±4.9)	0.95
Waist-to-hip ratio^a^	0.77	(±0.06)	0.77	(±0.06)	0.79	(±0.04)	0.33
Weight (kg)^a^,^b^	66.1		66.1		68.3		0.43
Breast volume (cm^3^)^a^,^b^	762		759		915		0.53

a The values for weight, waist-to-hip ratio and breast volume were measured 5–10 days prior to the predicted onset of the next menstrual period.

b The values for weight and breast volume were not normally distributed and the geometric means are presented.

**Table 2:** BRCA1 and BRCA2 mutation status of the cases and controls

	**All women (*n*=258)**	**Controls (*n*=252)**	**Cases (*n*=6)**
	**No. (%)**	**No. (%)**	**No.(%)**
*BRCA1*			
Positive	20 (7.8%)	16 (6.3%)	4 (80.0%)
Negative	47 (18.2%)	47 (18.7%)	—
Untested	17 (6.6%)	17 (6.7%)	—

*BRCA2*			
Positive	7 (2.7%)	7 (2.8%)	—
Negative	6 (2.3%)	6 (2.4%)	—
Untested	2 (0.8%)	2 (0.8%)	—

Non-BRCA1/2 family	113 (43.8%)	113 (44.8%)	—
Untested family	46 (17.8%)	44 (17.5%)	2 (20.0%)

We considered women who belong to known BRCA1 or BRCA2 families to be non-mutation carriers if they tested negative themselves or if their parent tested negative for the known mutation in the family. Untested women from BRCA1 and BRCA2 families have between 25 and 50% risk of carrying the mutation segregating in their respective families.

**Table 3:** Allele frequency distribution of polymorphic genetic variants by breast cancer status at follow-up

*IGF1* **genotype**	**All women (*n*=254)**	**No breast cancer (*n*=248)**	**Breast cancer (*n*=6)**
**(VNTR)**	**No. (%)**	**No. (%)**	**No. (%)**
12	3 (0.6%)	2 (0.4%)	1 (8.3%)
13	—	—	—
14	—	—	—
15	—	—	—
16	—	—	—
17	8 (1.6%)	8 (1.6%)	—
18	32 (6.3%)	32 (6.5%)	—
19	333 (65.6%)	330 (66.5%)	3 (25.0%)
20	91(17.9%)	87 (17.5%)	4 (33.3%)
21	35 (6.9%)	32 (6.5%)	3 (25.0%)
22	6 (1.2%)	5 (1.0%)	1 (8.3%)

*IGF1 19-repeat allele*			
Absent	33 (13.0%)	29 (11.7%)	4 (66.7%)
Present	221 (87.0%)	219 (88.3%)	2 (33.3%)

Genotype was missing for four women without breast cancer.
